# A novel nonparametric measure of explained variation for survival data with an easy graphical interpretation

**DOI:** 10.3205/000222

**Published:** 2015-10-29

**Authors:** Verena Weiß, Matthias Schmidt, Martin Hellmich

**Affiliations:** 1 Institute of Medical Statistics, Informatics and Epidemiology, University of Cologne, Germany; 2 Department of Nuclear Medicine, University of Cologne, Germany

**Keywords:** explained variation, survival data, novel measure, nonparametric, test of significance

## Abstract

**Introduction:** For survival data the coefficient of determination cannot be used to describe how good a model fits to the data. Therefore, several measures of explained variation for survival data have been proposed in recent years.

**Methods:** We analyse an existing measure of explained variation with regard to minimisation aspects and demonstrate that these are not fulfilled for the measure.

**Results:** In analogy to the least squares method from linear regression analysis we develop a novel measure for categorical covariates which is based only on the Kaplan-Meier estimator. Hence, the novel measure is a completely nonparametric measure with an easy graphical interpretation. For the novel measure different weighting possibilities are available and a statistical test of significance can be performed. Eventually, we apply the novel measure and further measures of explained variation to a dataset comprising persons with a histopathological papillary thyroid carcinoma.

**Conclusion:** We propose a novel measure of explained variation with a comprehensible derivation as well as a graphical interpretation, which may be used in further analyses with survival data.

## Zusammenfassung

**Einleitung:** Das Bestimmtheitsmaß kann bei Überlebenszeitdaten nicht verwendet werden um mithilfe einer Maßzahl anzugeben, wie gut ein Modell zu den vorliegenden Daten passt. Daher wurden in den letzten Jahren mehrere Maße der Erklärten Variation für Überlebenszeitdaten vorgeschlagen.

**Methoden:** Wir analysieren eines dieser Maße der Erklärten Variation bezüglich gewisser Minimierungseigenschaften und zeigen, dass diese für das Maß nicht erfüllt sind.

**Ergebnisse:** In Analogie zu der Kleinste-Quadrate-Methode aus der linearen Regression entwickeln wir ein neues Maß für kategorielle Kovariaten, welches nur auf dem Kaplan-Meier-Schätzer basiert. Dadurch ist das neue Maß komplett nichtparametrisch und besitzt eine einfache grafische Interpretation. Für das neue Maß stehen verschiedene Gewichtungsmöglichkeiten zur Verfügung und ein statistischer Signifikanztest kann angewendet werden. Abschließend bestimmen wir das neue Maß sowie weitere Maße der Erklärten Variation für die Personen eines Datensatzes mit einem histopathologisch gesicherten papillären Schilddrüsenkarzinom.

**Schlussfolgerung:** Wir schlagen ein neues Maß der Erklärten Variation mit einer eingängigen Herleitung sowie einer grafischen Interpretation vor, welches bei künftigen Auswertungen von Überlebenszeitdaten verwendet werden könnte.

## 1 Introduction

In linear regression analysis one often makes use of the coefficient of determination to describe how good a model fits to the data. The coefficient of determination is a measure with values between 0 and 1 and is defined as the square of the multiple correlation coefficient [1]. Due to censoring and a potential skewness of the data one cannot use this measure for survival data. Therefore, several measures of explained variation for survival data have been proposed in recent years, most of which are not easy to grasp or to interpret. In this work we present a novel measure of explained variation with a comprehensible derivation as well as a graphical interpretation. Furthermore we construct the novel measure in a completely nonparametric way and propose the application of a test of significance.

One of the measures of explained variation for survival data is the measure *V*_1_ by Schemper [2]. This measure has, together with the measure *V*_2_ by Schemper [2], been applied in several clinical, epidemiological and biological applications [3], [4], [5], [6]. In the measure *V*_1_ the impact of covariates is determined by the comparison of the distances of a singleton survival curve, i.e. the survival curve of only one person, to the Kaplan-Meier estimator of the entire group of persons and the distances of a singleton survival curve to the survival curve which results from a Cox proportional hazard model with given covariates. We analyse for Schempers measure *V*_1_ whether the distances employed in this measure are appropriate regarding minimisation aspects, which are chosen in analogy to the least squares method from linear regression analysis and the minimisation property of the median. In other words, we investigate whether *V*_1_ is based on a proper scoring rule [7], that again is based on a type of absolute error loss [8].

Based on the measure *V*_1_ by Schemper and the consideration of these minimisation aspects we derive a novel measure of explained variation 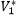 for categorical covariates with an easy graphical interpretation. On the one hand the novel measure defines the distance of a singleton survival curve to the Kaplan-Meier estimator in a different way and on the other hand it accounts for categorical covariates by using the distances of a singleton survival curve to the Kaplan-Meier estimator of the group to which the person belongs due to the covariates. The novel measure is based only on the Kaplan-Meier estimator and, as a consequence, is a completely nonparametric measure which can be depicted in a graphical way. Furthermore, different possibilities to weight the distances between a singleton survival curve and the Kaplan-Meier estimator at the event times are given and a statistical test of significance can be performed. This enables the comparison of the explained variation according to the novel measure 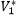 for two categorical covariates by inferential means. Finally, we apply the novel measure as well as further measures of explained variation to a dataset comprising 508 persons with differentiated papillary thyroid carcinoma.

## 2 Methods

### 2.1 The measure V_1_ by Schemper

The survival process of person *i* with *i* = 1, …,* n* at time *t_j_* with 1 ≤ *j* ≤ *k_i_* is given by *S_i_*(*t_j_*) and assumes a value of 1 if person *i* is at risk for the event at time *t_j_*, of 0.5 if *t_j_* is the exact failure time of person *i* and of 0 if person *i* has experienced the event before time *t_j_*. If person *i* experiences the event, the term *k_i_* is the total number of events in the sample. If person *i* is censored, *k_i_* is the number of events prior to the censoring of person *i*. The measure *V*_1_ by Schemper [2] is defined as 


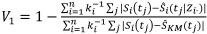
, 

where *Ŝ_KM_*(*t_j_*) denotes the Kaplan-Meier estimator at time *t_j_* and *Ŝ_i_*(*t_j_*|*Z_i·_*) the survival curve derived from a Cox model at time *t_j_* with covariates *Z_i·_* of person *i*. The measure *V*_1_ compares the distances between a singleton survival curve and the value of the Kaplan-Meier estimator at the event times *t_j_* (in the “denominator”) with the distances between a singleton survival curve and the value of the survival curve derived from a Cox model with given covariates (in the “numerator”) at the event times *t_j_*. The definition of the measure *V*_1_ by Schemper and Stare [9] is identical to the definition above with the exception that the survival process *S_i_*(*t_j_*) assumes a value of 1 if person *i* is at risk for the event at time *t_j_* and of 0 if person *i* has experienced the event before or experiences the event exactly at time *t_j_*. We will use this definition by Schemper and Stare throughout this paper.

### 2.2 Examination of minimisation aspects of the measure V_1_ by Schemper

To assess the relevance of the covariates the measure *V*_1_ by Schemper compares the distances between a singleton survival curve, i.e. the survival curve of only one person, and the value of the Kaplan-Meier estimator of the entire group of persons at the event times *t_j_* (in the denominator) with the distances between a singleton survival curve and the value of the survival curve derived from a Cox model with given covariates (in the numerator) at the event times *t_j_*. The distances to the value of the Kaplan-Meier estimator of the entire group of persons therefore serve as a reference for the relevance of the covariates. It would be desirable if in the denominator the vertical, absolute distances between the singleton survival curves and the value of the Kaplan-Meier estimator yield the Kaplan-Meier estimator as the optimal (minimising) step function, because the Kaplan-Meier estimator is a consistent estimator of the survival function with negligible bias [10]. Therefore it is desirable that the vertical, absolute distances generated in the denominator of *V*_1_ yield the Kaplan-Meier estimator as the optimal (minimising) step function. This is the case if at each event time the vertical, absolute distances above (“upper distances”) and below (“lower distances”) the Kaplan-Meier step function balance each other out, i.e. the sum of the distances above and the sum of the distances below the Kaplan-Meier step function have the same value at each event time. This approach arises from the minimisation property of the median, which implies that the median minimises the sum of the absolute differences from the single values to the median [11].

**Figure 1:**
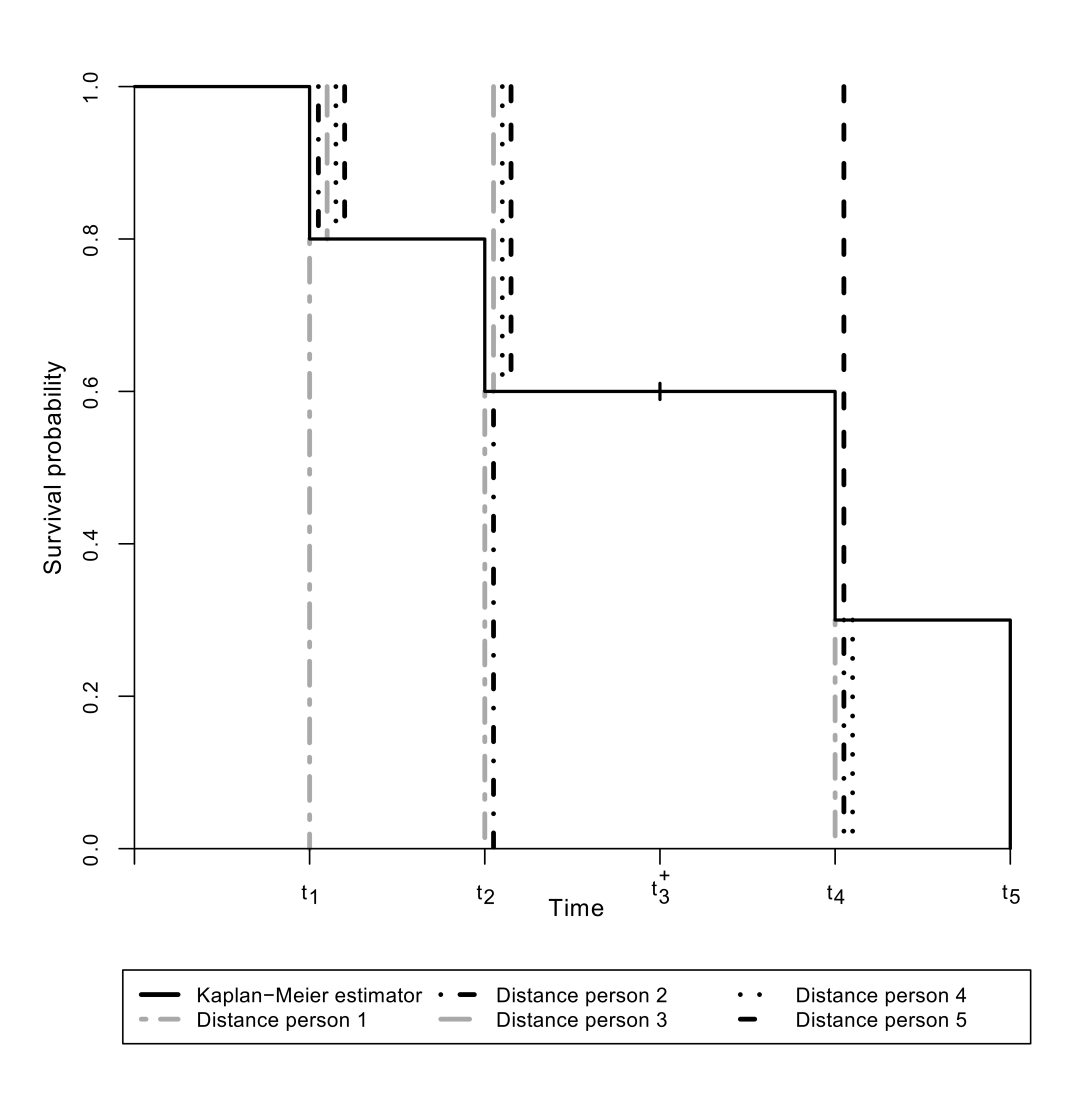
Distances in the measure *V*_1_ for the five persons in the example

To investigate this circumstance we set up a theoretical example with five persons: person 1 experiences the event at time *t*_1_, person 2 at time *t*_2_, person 3 is censored at time *t*_3_^+^, person 4 experiences the event at time *t*_4_ and person 5 at time *t*_5_. The vertical, absolute distances defined in the denominator of the measure *V*_1_ for the five persons are displayed in Figure 1 (Fig. 1). One can see that the upper distances for persons at risk at an event time reach from the value 1 to the value of the Kaplan-Meier estimator at the event time. Furthermore if a person experiences the event a lower distance at the event time itself as well as at the subsequent event times is given and reaches from the value 0 to the value of the Kaplan-Meier estimator at the respective event time. At the top of Table 1 (Tab. 1) the absolute values of these distances above and below the Kaplan-Meier step function are given for each person at every event time. At time *t*_5_ person 5 experiences the event and no person is at risk for the event. For this reason no upper distances are generated at this event time. The upper respectively lower distances are accumulated over the five persons at each event time. It can be seen that these accumulated upper and lower distances do balance each other out at the event times *t*_1_ and *t*_2_ in contrast to the event time *t*_4_. Therefore it can be stated that the distances in the measure *V*_1_ do not yield the Kaplan-Meier estimator as the optimal (minimising) step function at every event time, because the upper and lower distances do not balance each other out at every event time.

## 3 Results

### 3.1 Definition of a novel, nonparametric measure of explained variation V_1_^*^

**Figure 2:**
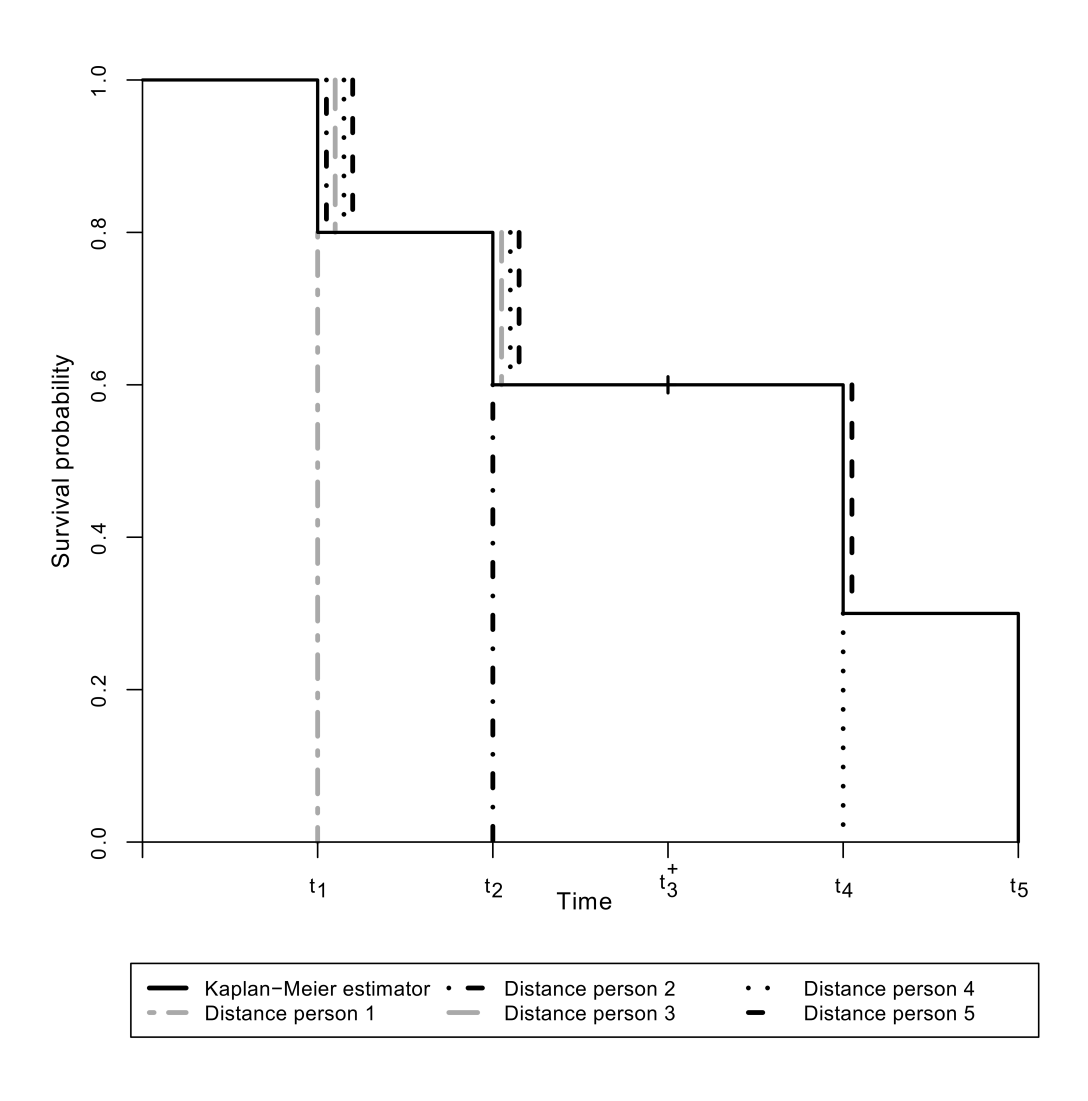
Distances in the measure *V*_1_^*^ for the five persons in the example

In this section a novel measure of explained variation 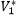 is developed. In this measure the vertical, absolute distances are defined in such a way, that they balance each other out at every event time. In the denominator of the novel measure 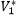 an upper distance at an event time *t_j_* for person *i* is defined as the vertical, absolute distance between the value of the Kaplan-Meier estimator at the preceding event time *t_j_*_–1_ and the value of the Kaplan-Meier estimator at the event time *t_j_* itself. In contrast to this each upper distance in the measure *V*_1_ starts at the value of the singleton survival curve of a person, i.e. at the value 1. Furthermore in the denominator of the novel measure 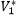 a lower distance is determined only at the event time *t_j_* of a person and reaches from 0 to the value of the Kaplan-Meier estimator at this event time. At the subsequent event times no lower distances are considered for the person in contrast to the measure *V*_1_. The upper and the lower distances in the novel measure 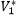 are analogous to the definition of the Kaplan-Meier estimator, in which the value of the estimation at an event time is dependent on the value of the estimation at the preceding event time and a person who experiences the event will not be considered in the estimation after the event time. Reconsidering the example with five persons, one can see that the vertical, absolute distances as defined in the novel measure 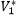 yield the Kaplan-Meier estimator as the optimal (minimising) step function at every event time. For this purpose the vertical, absolute distances defined in the measure 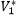 are illustrated for the five persons in Figure 2 (Fig. 2). For example, for the event time *t*_2_ it can be seen that the upper distances of the persons 3, 4 and 5, who are at risk for the event at this time, reach from the value of the Kaplan-Meier estimator at the preceding event time *t*_1_ to the value of the Kaplan-Meier estimator at time *t*_2_. Furthermore for persons experiencing the event, the lower distance contributes at the event time only. At subsequent event times no further lower distances are considered for these persons. At the bottom of Table 1 (Tab. 1) the values of the distances above and below the Kaplan-Meier estimator for each person at each event time are given and accumulated over the persons at each event time. In contrast to the measure *V*_1_ the accumulated upper and lower distances in the measure 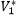 do balance each other out at every event time and, as a consequence, yield the Kaplan-Meier estimator as the optimal (minimising) step function.

We take a further modification of the measure *V*_1_ concerning the consideration of a categorical covariate *Z*_·1_ into account. In the numerator of the measure *V*_1_ by Schemper the impact of a covariate is assessed by the vertical, absolute distances of the singleton survival curve of person *i* to the survival curve yielded by a Cox model with given a covariate of the person. As a consequence the proportional hazards assumption must be satisfied for the measure *V*_1_. We modify the measure *V*_1_ by assessing the distances between the singleton survival curve of a person *i* to the value of the Kaplan-Meier estimator of the group, to which person *i* belongs according to the covariate. Therefore, the novel measure 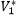 is a completely nonparametric measure, because it is based only on the Kaplan-Meier estimator.

The novel measure 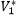 for a categorical covariate *Z*_·1_ is defined as


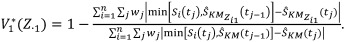


In this formula the term *S_i_*(*t_j_*) denotes the survival process of person *i* at time *t_j_*, *Ŝ_KM_*(*t_j_*) the Kaplan-Meier estimator of the entire group at time *t_j_* and 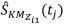 denotes the Kaplan-Meier estimator of the group to which person *i* belongs according to the covariate at time *t_j_*.

Due to the terms containing the minimum it is ensured that the upper respectively the lower distances to the Kaplan-Meier estimator of the entire group respectively of the group to which the person belongs according to the covariate *Z*_·1_ at time *t_j_* are provided. An upper distance thereby reaches from the value of the Kaplan-Meier estimator at the preceding event time *t_j_*_–1_ to the value of the Kaplan-Meier estimator at the event time *t_j_* and a lower distance from 0 to the value of the Kaplan-Meier estimator at the event time *t_j_*. For a person who experiences the event the index *j* contains all event times up to and including the event time of the person. For a censored person the index *j* contains all event times up to the censoring time of the person (including this time if another person experiences the event at this time). Furthermore, different weighting possibilities *w_j_* are available, which will be specified in detail in the next section.

### 3.2 Different weighting possibilities

The available weighting possibilities for the novel measure 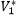 are analogous to the weighting possibilities of the Logrank test. It must be ensured that, despite the weighting term, the upper and lower distances balance each other out at every event time. The following weighting terms *w_j_* fulfil this requirement:

*w_j_* = 1: The vertical, absolute distances are weighted equally with the value 1 at every event time, which corresponds to the weighting in the Logrank test itself [12].


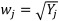
: The vertical, absolute distances are weighted with 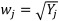, i.e. with the square root of the number of persons at risk for the event at the time *t_j_*. This weighting possibility corresponds to the weighting in the Tarone-Ware test [13].

*w_j_* = *Y_j_*: The vertical, absolute distances are weighted with *Y_j_*, i.e. with the number of persons at risk for the event at the time *t_j_*. This weighting possibility corresponds to the weighting in the Breslow test [12].

In the last two weighting possibilities the vertical, absolute distances at the beginning of the study, i.e. at times with a larger number of persons at risk, are more emphasized than the vertical, absolute distances at the end of the study, i.e. at times with fewer persons at risk.

### 3.3 Test of significance

In this section we suggest how the explained variation of a covariate *Z*_·1_ can be compared to the explained variation of a covariate *Z*_·2_ by inferential means. Assuming that the two covariates are gathered from the same persons, the test of significance has to be a test for paired samples. In the previous section we noted that the novel measure of explained variation 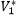 is a completely nonparametric measure and consequently the corresponding test of significance should be nonparametric as well. Therefore we apply the Wilcoxon test for paired samples [14].

As can be detected in the formula, the covariates are accounted for solely in the numerator of the measure 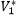. For the covariates *Z*_·1_ and *Z*_·2_ the corresponding terms in the numerator, i.e. the accumulated weighted sum of distances of a person i according to the covariate *Z*_·1_ and *Z*_·2_ respectively, are 


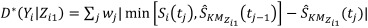
 and


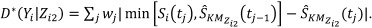


To compare two covariates due to their extent of explained variation, a Wilcoxon test for paired samples is performed over the differences of these accumulated distances, i.e. over the terms D_i_^*^ = D^*^ (*Y_i_*|*Z_i_*_2_ ) – D^*^(*Y_i_* |*Z_i_*_1_).

### 3.4 Medical application

We apply the novel measure 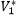 as well as other measures of explained variation to a dataset comprising 508 persons with a histopathological papillary thyroid carcinoma. These persons were treated by total thyroidectomy and subsequent radioiodine therapy with 3.7 GBq between Januar 1990 and June 2005 at the Department of Nuclear Medicine, University Hospital of Cologne in Germany. The tumour of each person was retrospectively classified according to the fifth [15], the sixth [16] and the seventh version [17] of the UICC tumour classification system. The UICC classification system describes the anatomical extent of a malignant disease and is based on the assessment of three components. The first component “T” describes the extent of the primary tumour, the second component “N” the absence or presence and extent of regional lymph node metastases and the third component “M” the absence or presence of distant metastases. Between the three versions of the UICC tumour classification system the margins, which assign a thyroid carcinoma to the respective tumour category of component “T”, were redefined. More precisely the margins for T1 tumours were extended from 1 centimetre in the fifth version to 2 centimetres in the sixth version. Furthermore a minimal extrathyroidal growth, which defines a T4 tumour in the fifth version, defines a T3 tumour in the sixth version. In the seventh version the margins of the sixth version were not changed, but T1 tumours are subdivided into the categories a and b, for tumour diameters ≤1 centimetre respectively >1 centimetre. As a consequence the fifth and sixth version each comprise four categories, whereas the seventh version consists of five categories. Based on this dataset we seek to answer the question whether the fifth, the sixth or the seventh version of the UICC classification system explains the most variation for the outcome variable “time to the occurrence of a distant metastasis”. In the 508 persons with a papillary thyroid carcinoma 25 distant metastases were diagnosed. A detailed description of the dataset, which comprises as a whole 636 persons with either a papillary or a follicular thyroid carcinoma, can be found in Meixner et al. [18]. Since only routinely collected data were retrospectively evaluated ethical review was not required.

The allocation of the tumours of the 508 persons with a papillary thyroid carcinoma to the categories according to the fifth and the sixth version of the UICC tumour classification system is given in Table 2 (Tab. 2). One can see, that the tumour of 227 persons is assigned to different categories by the fifth respectively the sixth version. The seventh version subdivides the tumours allocated to the category pT1 by the sixth version merely into the categories pT1a (133 tumours) and pT1b (127 tumours). The 25 events occur as follows in the categories pT1 (respectively pT1a and pT1b), pT2, pT3 and pT4: 2, 6, 3, 14 events (fifth version), 4, 4, 8, 9 events (sixth version) and 2, 2, 4, 8, 9 events (seventh version). To analyse whether the fifth or the sixth version explains more variation, the novel measure 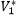 with weighting term *w_j_*=1 is calculated on the one hand with the fifth version as a covariate and on the other hand with the sixth version as a covariate. For the time to the occurrence of a distant metastasis the measure 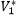 has a value of 0.0239 for the fifth version and of 0.0695 for the sixth version of the UICC tumour classification system. The corresponding test of significance, i.e. the Wilcoxon test for paired samples, has a p-value less than 0.0001. In summary the explained variation has a larger value according to the sixth version than the explained variation according to the fifth version for the outcome variable and the difference is statistically significant at the significance level α=0.05. Consequently it can be stated that the sixth version explains significantly more variation than the fifth version of the UICC tumour classification system for the outcome variable. For the seventh version the measure 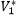 has a value of 0.0700 and explains in comparison to the sixth version significantly more variation (*p*<0.0001). To put the results of the measure 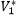 in a broader context the values of further measures of explained variation are considered, these are the measures *V*_1_ and *V*_2_ by Schemper [2], the measure *V* by Schemper and Henderson [19], a measure 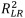 based on the likelihood ratio test [20], the measure 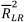 by Nagelkerke [21] and the measure 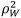 by Kent and O’Quigley [22] as well as the approximation thereof 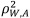 [22]. To compute the measures of explained variation the statistic software packages R 2.15.1 (measures 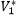, 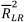), SAS^®^ 9.3 (TS1M0) with SAS/STAT 9.3 (measures *V*_1_, *V*_2_, 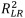, 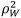, 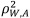) and SAS^®^ 9.4 (TS1M1) with SAS/STAT 13.1 (measure *V*) are used [23], [24], [25]. Details concerning the computation of the measures can be found in the Appendix.

The values of the measures of explained variation for the outcome variable with the fifth, the sixth and the seventh version of the UICC tumour classification system respectively, as a covariate are given in Table 3 (Tab. 3). The values of the measure 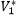 and the measure *V*_1_ are of a quite similar dimension, as well as the values of the measure *V*_2_, which are just slightly smaller than the values of the measures 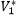 and *V*_1_. Contrarily, the values of the measures *V* and 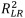 are predominantly larger, followed by the values of the measure 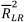. The values of the measures by Kent and O’Quigley 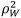 and 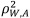 are considerably larger than the values of all other measures of explained variation. Furthermore one can see that for all measures of explained variation the values according to the sixth version are larger than the values according to the fifth version. The values according to the seventh version are, in comparison to the values of the sixth version, identical or slightly larger.

## 4 Discussion

The novel measure of explained variation 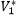 is a comprehensible measure which can be depicted in a graphical way. As a reference for the relevance of a categorical covariate the vertical, absolute distances between the singleton survival curves and the value of the Kaplan-Meier estimator are defined in this measure in such a way that the distances above and below the Kaplan-Meier estimator balance each other out at every event time. This property is desirable in analogy to the least squares method from linear regression analysis and the minimisation property of the median. Furthermore in the measure 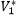 the explained variation is determined by the comparison of these distances to the Kaplan-Meier estimator of the group to which the person according to the covariate belongs. Thereby the novel measure is based only on the Kaplan-Meier estimator and, as a consequence, is a completely nonparametric measure. The sole assumption is the validity of the “independent censoring assumption”, which requires that the censored persons at a time are representative for the persons at risk at this time [26]. The interpretation of the distances applied in the measure 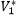 arises from the analogy to the definition of the Kaplan-Meier estimator, in which the value of the estimation at an event time is dependent on the value of the estimation at the preceding event time and a person who experiences the event will not be considered in the estimation after the event time. Furthermore, different weighting possibilities are available for the measure 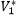. These are comparable to the weighting possibilities of the Logrank test. One can therewith weight the distances at each event time equally or one can weight the distances at the early event times with many persons at risk more strongly than the event times at the end of the study with fewer persons at risk.

A further aspect of this work is the application of the Wilcoxon test for paired samples to compare the explained variation of two covariates. In addition to the mere comparison of the values of the measure 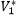 it can be analysed whether one covariate explains significantly more variation than the other covariate, i.e. whether the difference is statistically significant or not. This significance test could be implemented for the measure *V*_1_ as well. However, for both measures the assumptions required for the Wilcoxon test for paired samples are not entirely met (i.e. the independence of paired differences may be violated due to reference to a common Kaplan-Meier curve), respectively need to be assumed (symmetric continuous distribution of the paired differences around the median 0). The latter assumption is not required for the exact binomial sign test, while both requirements can be waived for an appropriate permutation test.

Further limitations of the measure 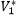 must be stated. Continuous variables have to be categorised, which entails a loss of information, before being incorporated in the measure 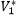. Note that an upper limit for the number of categories does not exist, i.e. the measure can always be calculated. However in order to get valid and reproducible results the “10 events per variable/category rule” may be applied [27]. In the extreme case where every person is in his or her own category the explained variation would amount to 1. Furthermore the quantity which is supposed to be estimated by the novel measure, i.e. the estimand [28], is hard to grasp. However the novel measure 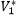 has the following advantages: It is a proper scoring rule, which yields the Kaplan-Meier estimator as the optimal minimising step function at every event time. Afterwards the measure combines the respective distances over all event times with the consequence that it does not solely correspond to a single event time. Furthermore the measure has a comprehensible derivation with a graphical interpretation and is a completely nonparametric measure. Moreover the covariates in the medical application, which is described in this manuscript, are categorical. The possibility to incorporate continuous covariates into the measure is not needed for this medical application.

In contrast to the measure 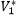, the distances above and below the Kaplan-Meier estimator in the measure *V*_1_ do not balance each other out at every event time. The same is true for the measure *V*_2_, which solely differs from the measure *V*_1_ by taking the square of the summed and averaged distances. The measures *V*_1_, *V*_2_ and *V* each determine the explained variation by comparing the distances of a singleton survival curve to the survival curve derived from a Cox model with given covariates. The Cox model is based on the assumption of proportional hazards, which therefore should be checked prior to application. This is not required for the novel measure 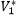. However, it might be possible to compute the measures *V*_1_, *V*_2_ and *V* also with other survival models than the Cox model to avoid the assumption of proportional hazards.

Furthermore, the persons who are censored prior to the first event time need to be removed from the dataset prior to the computation of the measures *V*_1_ and *V*_2_. In these measures the weighting term for a censored person is given by the number of events prior to the censoring of a person, which is 0 for these persons. This results in an impossible division by 0 for the persons, who are censored prior to the first event time. In contrast to this in the novel measure 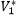 the weighting term is depicted in a different way, with the consequence that the persons, who are censored prior to the first event time, can remain in the dataset.

As mentioned in the introduction, several measures of explained variation for survival data have been proposed in recent years. The measures applied in this work either follow a “distance-based” or a “likelihood-based” approach. In a “distance-based” approach a scoring rule measures the distance between predicted and observed survival outcome. A further popular measure in this field is the Brier score, which is calculated at fixed time points and afterwards combined to give a time-dependent curve [29], [30], [31]. In a “likelihood-based” approach a measure relates the log likelihood of a model with covariate information to the log likelihood obtained from a “null model” ignoring covariate information [29]. Furthermore many measures have been proposed, which follow a “discrimination-based” approach. These measures are popular tools to characterise the predictive performance of a survival model and use this model to distinguish between persons having an event and persons having no event at any specific time point. For example, the time-dependent area under the ROC curve can be used as a measure of the discriminative ability of the survival model at each time point [29], [30].

Further research could be performed for the novel measure 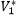, which is proposed in this work. One step would be the construction of a confidence interval for the measure itself as well as the construction of a confidence interval for the mean difference of the explained variation of two covariates. Furthermore it would be important to determine the maximum value for the measure 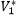.

The measure *V*_2_ as well as the measure 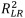 are bounded above by a constant less than 1 [21], [32]. For the measure 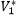 the maximum value in a dataset is unknown. Theoretically the measure has the maximum value of 1, if the number of categories is equal to the number of event times in a dataset. This is for example the case if every person is in his or her own category. Furthermore this is the case if several persons are in the same category and these persons experience the event at the exact same time or if some of these persons experience the event at the exact same time and the others are censored. Contrarily, if the number of categories is smaller than the number of event times, at least one category exists in which the persons experience the event at different event times. In this latter case the measure 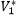 cannot gain the maximum value of 1. Therefore it might be sensible to depict the maximum value of the measure 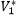 in a given dataset. Afterwards the value of the measure could be standardised to this maximum value. For a limited number of persons the exact maximum value could be identified by a simulation of all possible permutations of the number of events, the number of censorings as well as the number of categories. For a large number of persons an approximation thereof would be feasible. The functions to compute the novel measure 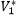 and to apply the test of significance can be obtained on request. The circumstance that some measures of explained variation cannot gain the maximum value of 1 might be responsible for the huge range of values in the measures of explained variation in the dataset of persons with histopathological papillary thyroid cancer. Based on this dataset we seek to answer the question whether the fifth, the sixth or the seventh version of the UICC classification system is the “better” classification system for the time to the occurrence of a distant metastasis. A traditional medical approach to answer the question concerning the “best” version of the UICC classification system would be a prospective randomised clinical trial. A person would be randomised to the fifth, the sixth or the seventh version of the UICC classification system and the tumour would be categorised accordingly. At the end of the study the time to the occurrence of the outcome variable could be compared for the versions of the UICC classification system. However, for persons with thyroid cancer, the performance of a prospective randomised clinical trial is not feasible in this context due to different reasons. First of all a differentiated thyroid tumour is a rare disease with an usually slow natural progression [18]. Furthermore different versions of the UICC tumour classification system are not valid at the same time, rather replaces an updated version the previous version.

Therefore, to answer the question which version of the UICC classification system is the “best” version, we compare the versions according to their proportion of explained variation and the “best” version is the version which explains the most variation. In spite of the huge range of values of the measures of explained variation for the persons with histopathological papillary thyroid cancer, all measures have in common that the sixth version explains more variation than the fifth version of the UICC tumour classification system for the outcome variable. The values of the measures of explained variation according to the seventh version are, in comparison to the values of the sixth version, identical or slightly larger. However, one has to keep in mind, that the fifth and the sixth version each comprise four categories whereas the seventh version consists of five categories. The gain in the values of the measures of explained variation for the seventh version in comparison to the values of the sixth version might be induced by the existence of an additional category in the seventh version. A similar circumstance is known for the coefficient of determination where an increasing number of predictors usually leads to an increase in the coefficient of determination, even when the true values of the new regression coefficients are zero [33]. Therefore, the comparison of the fifth and the sixth version of the UICC tumour classification system might be more appropriate than the comparison of the sixth and the seventh version. It can be stated that the sixth version of the UICC tumour classification system is “better” than the fifth version with respect to the amount of explained variation for the outcome variable “time to the occurrence of a distant metastasis” in the dataset of persons with histopathological papillary thyroid cancer.

## 5 Conclusion

The novel measure of explained variation proposed in this work is a measure with a comprehensible derivation as well as a graphical interpretation. It is based only on the Kaplan-Meier estimator and is therefore a completely nonparametric measure. Furthermore, we propose the application of a statistical test of significance additionally to the computation of the measure itself. The novel measure may for categorical covariates be used as a measure of explained variation in further analyses with survival data and the obtained results might additionally be evaluated by inferential means.

## Data

Data for this article are available from the Dryad Digital Repository: http://dx.doi.org/10.5061/dryad.5c6bq [34].

## Notes

### Competing interests

The authors declare that they have no conflicts of interest in the research.

## Appendix

Details for the computation of the measures of explained variation in subsection 3.4 are given below.

The functions to compute the novel measure 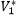 and to apply the corresponding test of significance are developed for the software package R 2.15.1 [23] and can be obtained on request. The computations of the measures 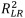 [20] and 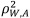 [22] are directly carried out in SAS^®^ 9.3 (TS1M0) with SAS/STAT 9.3 [24] according to the definition of the measures. For the remaining measures the following programs, functions and options are applied: Lachin [32] provides on the website accompanying the first edition of his book (http://www.bsc.gwu.edu/bsc/webpage.php?no=18 (last accessed 03/09/2015)) a program for the computation of the measure *V*_2_ by Schemper [2]. This program is subject to minor changes prior to application: the term *k_i_* is altered to the number of events in analogy to the definition by Schemper, whereas in the program by Lachin the term *k_i_* is defined as the number of event times. Furthermore the squaring in the measure *V*_2_ is positioned at the correct spot, the covariates are considered as categorical variables in the respective Cox model and the persons who are censored prior to the first event time are removed from the dataset. To compute the measure *V*_1_ by Schemper [2] this program is extended. For the computation of the measures *V*_1_ and *V*_2_ the software package SAS^®^ 9.3 (TS1M0) with SAS/STAT 9.3 [24] is used. The measure *V* by Schemper and Henderson [19] is determined with the option “EV” in the procedure “PHREG” in SAS^®^ 9.4 (TS1M1) with SAS/STAT 13.1 [25], the measure 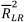 [21] with the function “cph”, package “rms” in R 2.15.1 [23] and the measure 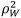 [22] with the macro “KENTOQNR” by Heinzl [35] in SAS 9.3^®^ (TS1M0) with SAS/STAT 9.3 [24]. For all measures, which are based on the computation of the likelihood function, the approximation by Breslow [36] is used.

## Figures and Tables

**Table 1:**
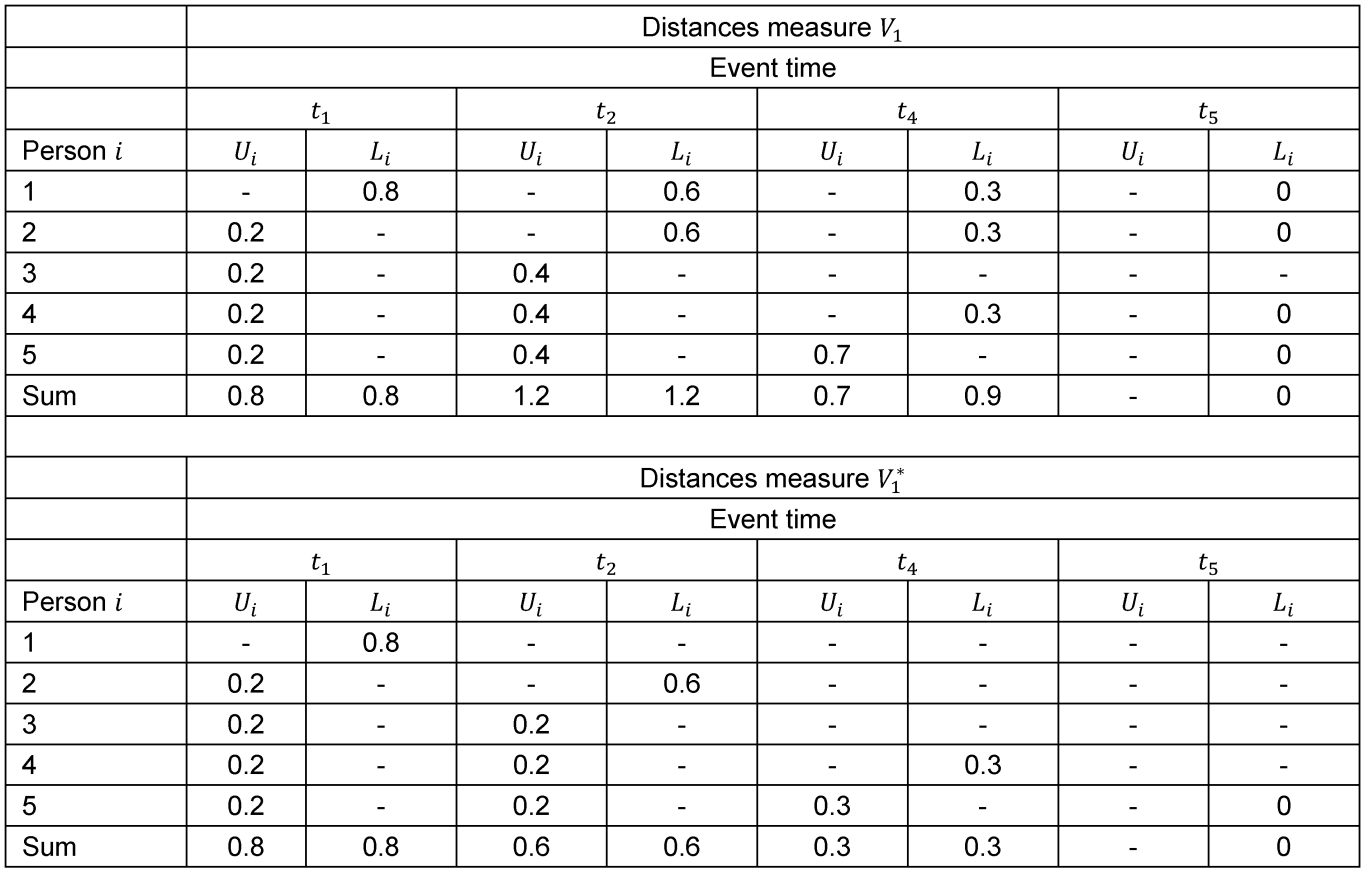
Distances above (*U_i_*) and below (*L_i_*) the Kaplan-Meier estimator in the measures *V*_1_ and *V*_1_^*^ in the example

**Table 2:**
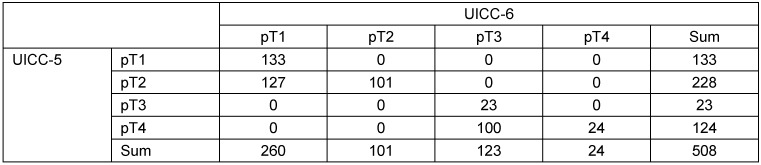
Allocation of the tumours to the categories of the fifth and sixth version of the UICC tumour classification system

**Table 3:**
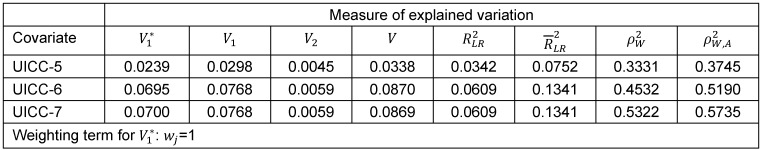
Values of various measures of explained variation considering one of three versions of the UICC tumour classification system as a covariate for the outcome variable
